# Trajectories of disability in low back pain

**DOI:** 10.1097/PR9.0000000000000985

**Published:** 2022-01-17

**Authors:** Tonny E. Andersen, Karen-Inge Karstoft, Henrik H. Lauridsen, Claus Manniche

**Affiliations:** a Department of Psychology, University of Southern Denmark, Odense, Denmark; b Department of Psychology, University of Copenhagen, Copenhagen, Denmark; c Department of Sports Science and Clinical Biomechanics, University of Southern Denmark, Odense, Denmark; d Department of Occupational and Environmental Medicine, Odense University Hospital, Institute of Clinical Research, University of Southern Denmark, Odense, Denmark

**Keywords:** Low back pain, Disability, Recovery, Trajectories, Latent growth mixture modeling

## Abstract

**Introduction:**

Low back pain (LBP) is the leading course of years lived with disability. Unfortunately, not much knowledge exists about distinct trajectories of recovery from disability after LBP and their potential psychological predictors.

**Objectives:**

Hence, the aim of the present study was to identify trajectories of functional disability in LBP and their potential baseline psychological predictors.

**Methods:**

A 1-year consecutive cohort (N = 1048) of patients with LBP referred to the Spine Centre if they have not improved satisfactorily from a course of treatment in primary care after 1 to 2 months were assessed by self-report questionnaires at their first visit and at 6- and 12-month follow-up. Data from patients who responded to the Roland Morris Disability Questionnaire at least twice (N = 747) were used to assess trajectories of functional disability by Latent Growth Mixture Modeling. The following measures were used as baseline predictors of the trajectories: Pain Intensity Numerical Rating Scales, Pain Catastrophizing Scale, Tampa Scale for Kinesiophobia, and Hospital Anxiety and Depression Scale.

**Results:**

Four distinct trajectories were identified: high-stable (22.0%), high-decreasing (20.4%), medium-stable (29.7%), and low-decreasing (27.9%). Using the low-decreasing trajectory as reference, baseline pain intensity, depressive symptoms, and pain-catastrophizing predicted membership of all 3 symptomatic trajectories. However, using the high-decreasing trajectory as reference, age, baseline pain intensity, and depression were predictors of the high-stable trajectory.

**Conclusion:**

In particular, the finding of a high-stable trajectory characterized by high levels of baseline psychological distress is of potential clinical importance because psychological distress may be targeted by cognitive behavioral therapeutic approaches.

## Abstract 2

Four recovery trajectories from low back pain were identified: high-stable (20.9%), high-decreasing (20.7%), medium-stable (29.9%), and low-decreasing (28.5%). Pain and depression predicted the high-stable trajectory.

## 1. Introduction

Low back pain (LBP) is the most common type of musculoskeletal pain with an estimated lifetime prevalence of up to 80%.^15^ More importantly, epidemiologic studies find that LBP is the leading course of years lived with disability.^14^ Unfortunately, diagnostic information is a poor predictor of recovery.^38^ However, a stratified care approach, stratifying high-risk patients to psychologically augmented physiotherapy, has been shown to result in significantly better recovery from disability compared with current best primary care.^12,16^ Without such stratification, many low-risk patients receive unnecessary treatment, whereas high-risk patients may not be offered the right treatment targeting psychological risk factors.^17^ Hence, more knowledge about distinct trajectories of recovery after LBP and their potential predictors is needed for better targeting of interventions to those in need. While trajectories of pain and disability may develop in parallel, their psychological predictors may be different, which is important from a clinical perspective. For instance, when eradication of pain is not an option, elimination of psychosocial barriers that contribute to the development and maintenance of disability may still be targeted in pain rehabilitation.

Although a number of studies have assessed trajectories of pain in LBP,^5,7–16,22^ fewer studies have assessed trajectories of disability.^6,9^ Only Deyo et al.^9^ aimed to identify subgroups and this was in older adults (>65 years) with no psychological predictors in the models. On average, patients improved <10% in disability. Five distinct trajectories of functional recovery were recognized, of which one small but substantial subgroup of 6.1% was characterized by initially high levels of disability with substantial functional improvement of 81%. Importantly, another highly stable trajectory (18.5%) was identified with equally high levels of disability at baseline but with no improvement over time. The other 3 trajectories were characterized by different but relatively stable functional limitations. Comorbidity, pain duration, and confidence in recovery were significantly associated with recovery.^9^

Estimating trajectories of recovery from LBP, 4 to 5 trajectories are often identified.^20^ A subgroup following a persistent severe trajectory is characterized by initial high levels of pain intensity with no recovery over time and a trajectory of ongoing or fluctuating moderate levels of pain intensity and finally a recovery trajectory (for a review see 20). Most recently, Chen et al.^8^ confirmed the 4 trajectories of recovery from pain^11^ and found levels of pain-catastrophizing, fear of movement, anxiety, and depression to be associated with lack of recovery from pain intensity.^8^ Similar trajectories of recovery from pain intensity have been described in whiplash cohorts.^3,39^

To the best of our knowledge, no studies have assessed trajectories of disability and their psychological predictors in a representative cohort of patients with LBP. Hence, the aim of the present study was to identify trajectories of disability after LBP and test baseline psychological predictors of potential trajectories. First, we hypothesized to find distinct trajectories of disability, here among a chronic nonrecovering class. Second, we hypothesized that gender, age, and baseline levels of pain intensity, pain-catastrophizing, fear-avoidance beliefs (kinesiophobia), and depressive symptoms would be predictive of class membership.

## 2. Materials and methods

### 2.1. Participants and procedures

A longitudinal cohort design was used to investigate the hypotheses. Questionnaire data were collected consecutively between January 2013 and August 2014 from a 1-year cohort (N = 1048) of patients referred to a large Danish Spine Centre. Patients were recruited to the present study as part of the standard screening procedure at admission to the Spine Centre. Follow-up questionnaires were sent at 6- and 12-month follow-up by post. Two reminders were sent after 2 and 3 weeks if questionnaires were not returned. Patients were referred to the Spine Centre if their primary complaint was related to the spine and if their pain symptoms had not improved satisfactorily from a course of treatment in primary care after 1 to 2 months. Only patients with no indications for acute spinal surgical intervention were included. Patients with severe diseases such as cancer, infections, or inflammatory spondylopathies were excluded.

The Spine Centre is a government-funded hospital with a large catchment area of approximately 1.2 million people (for a more detailed description see 18). The study was approved by the Danish Data Protection Agency, and the review board of University of Southern Denmark approved the research protocol. All patients gave informed written consent.

### 2.2. Measures

Disability was measured with the modified version of the Roland Morris Disability Questionnaire (RMDQ-23).^31^ The RMDQ-23 contains 23 items measuring activity limitation associated with back and leg pain. Each item is dichotomously scored (yes/no) according to the affirmation of an item statement associated with activity limitation “today.” Test–retest reliability, internal consistency, and construct validity of the Danish version have been found to be good.^2^

Pain intensity was measured as the average score of three 11-point Likert scales measuring peak pain intensity, average pain intensity over the past 2 weeks, as well as current pain intensity. Each scale measures pain intensity on a 0 to 10 numerical rating scale, with 0 defined as no pain and 10 as the worst imaginable pain. The scale has good internal consistency, construct, and predictive validity.^24^

The Pain Catastrophizing Scale (PCS)^40^ was used to measure catastrophic thinking related to pain. The PCS asks participants to reflect on past painful experiences and to indicate the degree to which they experienced each of 13 thoughts or feelings when experiencing pain, on a 5-point Likert scale (0 = not at all and 4 = all the time). A total scale score ranging from 0 to 52 was calculated from all items, with a high score indicating a high level of pain-catastrophizing. The Danish version of the PCS has been validated in both a clinical and a nonclinical sample and has good internal consistency, construct, and predictive validity.^19^

Fear of reinjury due to movement was measured with the Tampa Scale for Kinesiophobia.^21^ Tampa Scale for Kinesiophobia is a 17-item scale assessing fear of movement on a 4-point Likert scale ranging from 17 to 68, with higher scores indicating higher levels of kinesiophobia. The scale is commonly used in diverse chronic pain samples and has good internal consistency, construct, and predictive validity.^34^

Levels of depressive symptoms and anxiety were measured with the Hospital Anxiety and Depression Scale.^43^ The depression scale consists of 7 items related to depression, and the anxiety scale consists of 7 items related to anxiety. Symptom levels on both subscales are measured on a 4-point Likert scale ranging from 0 to 21, with a high score indicating high levels of depression and anxiety.

### 2.3. Statistical analyses

Trajectories of disability were estimated by Latent Growth Mixture Modeling (LGMM). No assumptions about the number or characteristics of such trajectories were made a priori, and hence, the analysis was conducted in a data-driven fashion. In LGMM, the starting point is the estimation of a model with one trajectory, encompassing the entire sample. For this trajectory, intercept and slope is estimated. Subsequently, models are estimated with an increasing number of trajectories, continuing until convergence can no longer be reached. Selecting the final model depends largely on model fit, which is based on fit indices and likelihood ratio tests, where the model with n classes is directly compared to the model with n − 1 classes.^28^ However, fit indices and likelihood ratio tests might not provide conclusive answers, and hence, clinical and theoretical meaningfulness as well as parsimony of the model need also be taken into account. Because we had data for 3 time points only, we were only able to estimate linear and not quadratic growth.

More specifically, we applied the Akaike Information Criterion,^1^ the Bayesian Information Criterion,^36^ and the sample size–adjusted Bayesian Information Criterion, as well as the Lo–Mendell–Rubin Likelihood Ratio Test (LMR-LRT)^22^ and the bootstrapped LRT (BLRT)^25^ for evaluating the fit of the model. With the establishment of a model, it is a common wish to explore predictors or covariates of class membership, which was also the case in our study. Such testing of covariates can be done in different ways: by inclusion of covariates in the model,^41^ by using a 3-step approach as described by Vermunt,^42^ or by testing covariates in a post hoc fashion outside the model.^41^ Selecting the appropriate method depends on results of the trajectory analysis, most importantly the entropy of the model. Preempting our results, we found acceptable but low entropy. This calls for inclusion of covariates in the model with the 2-fold aim of (1) increasing classification accuracy and (2) avoiding biased estimates that can arise in post hoc covariate analysis, where class membership is treated as a set entity instead of the estimate it rightfully is.^41^ Hence, after model selection, we included covariates of interest in the model, performing a multinomial logistic regression with class membership as the dependent variable. Independent variables of interest included sex, age, and baseline levels of pain intensity, kinesiophobia, pain-catastrophizing, anxiety, and depression. All analyses were conducted in Mplus version 8.^26^

### 2.4. Handling of missing data

For the main LGMM analyses, we included only individuals who responded to the RMDQ at least twice (N = 747). Applying a criterion of response to all 3 assessments would result in a relatively large proportion being listwise deleted, leading to potential bias (missing at T1: n = 7, missing at T2: n = 132, missing at T3: 188). Hence, we settled on participation at 2 assessments as the inclusion criteria and used full information likelihood (FIML) estimation to account for the missing data points. With inclusion of covariates in the model, only individuals with full data on all covariates were included even with FIML. Hence, to once again avoid massive listwise deletion, we applied multiple imputation of covariates,^4^ enabling us to include all 747 individuals in the analysis. For most covariates, low rates of missing were seen (pain intensity: 1.2%, depression: 4.4%, anxiety: 5.9%, pain-catastrophizing: 10.8%); however, for kinesiophobia, the rate of missing was 22.6%. Imputation was conducted in Mplus using the default Bayesian method.^35^ Ten datasets were created and results from the pooled analysis reported.

## 3. Results

### 3.1. Participants

Of the 1048 patients, 747 responded at least twice. At baseline, the mean age for the patients was 47.55 years (SD *=* 10.95). In total 57% were female. In relation to age, sex, pain intensity, and disability symptoms, the included sample was representative of the general cohort referred to the spine center (for a description of the cohort, see 18). In Table 1, baseline characteristics of the overall sample and the members of the 4 trajectories are presented.

**Table 1:** Demographics and baseline characteristics of the overall sample and trajectories.

	All N = 747	Trajectories				Group difference		
		High-stable (22.0%)	High-decreasing (20.4%)	Medium-stable (29.7%)	Low-decreasing (27.9%)	X^2^/F	*df*	*P*
Females (%)	57.0	56.1	51.5	67.5	52.8	12.6	3	0.006
Mean age (SD), y	47.6 (10.9)	48.9 (10.3)	46.6 (11.1)	48.7 (10.9)	46.0 (11.2)	3.4	3	0.017
Work situation (%)						86.6	24	<0.001
No change in work situation	59.6	40.9	70.4	56.3	70.2			
Unemployed	5.5	7.9	1.9	6.0	5.8			
Sick leave	4.2	2.4	4.4	3.5	6.3			
Work easier	5.6	7.3	6.3	7.0	2.1			
Reduced hours	8.8	17.7	7.5	7.5	3.7			
Subsidized job	1.7	4.9	0	1.5	0.5			
Use of analgesics (%)	71.7	88.1	78.8	73.0	50.3	72.1	3	<0.001
Paracetamol	63.9	75.0	73.3	63.3	47.6	32.9	3	<0.001
Acetylsalicylic acid	2.4	3.0	2.6	1.9	2.4	0.3	3	0.951
NSAID	50.3	56.1	64.2	47.7	37.4	24.1	3	<0.001
Codeine	5.0	8.8	5.0	2.5	4.3	6.3	3	0.097
Tramadol	21.5	33.8	29.3	22.0	4.8	44.2	3	<0.001
Morphine	6.6	16.2	5.9	5.1	0.6	30.4	3	<0.001
Other analgesics	19.3	32.6	17.5	19.0	9.6	26.5	3	<0.001

NSAID, nonsteroidal anti-inflammatory drugs.

The distribution of age and gender across the members of the 4 trajectories were small but statistically different. Most of the members of the stable trajectories were females and 2 to 3 years older than members of the decreasing trajectories.

Only 40.9% members of the high-stable trajectory had not experienced a change in their work situation after onset of back pain compared with the 70.2% members of the low-decreasing trajectory. Compared to all other trajectories, more in the high-stable trajectory were in a subsidized job or on reduced work time. Also, the high-stable trajectory group was characterized by a higher use of pain medication compared with the other trajectory groups. For more details, see Table 1.

### 3.2. Trajectories of disability symptoms

Fit indices of all models can be seen in Table 2. We present fit indices of the model *before* imputation and inclusion of covariates since the final model was selected before including covariates. To aid model convergence, we ran models where in-group variance of the slope was fixed to zero while intercept was estimated freely.

**Table 2:** Fit indices of 1 to 6 class models.

	AIC	BIC	Ssa-BIC	LMR-LRT	BLRT	Entropy
1 class	17,462.11	17,489.81	17,470.76			
2 classes	17,275.49	17,317.04	17,288.46	*P* < 0.001	*P* < 0.001	0.70
3 classes	17,226.73	17,282.12	17,244.01	*P* < 0.001	*P* < 0.001	0.66
4 classes	17,197.37	17,266.62	17,218.98	*P* < 0.001	*P* < 0.001	0.65
5 classes	17,185.21	17,268.30	17,211.14	*P* = 0.059	*P* < 0.001	0.65
6 classes	17,170.26	17,267.20	17,200.52	*P* = 0.211	*P* < 0.001	0.65

AIC, Akaike Information Criterion; BIC, Bayesian Information Criterion; BLRT, bootstrapped LRT; LMR-LRT, Lo–Mendell–Rubin Likelihood Ratio Test; ssa-BIC, sample size–adjusted BIC.

By scrutinizing fit indices and likelihood ratio tests, we settled on the four-class model as the optimal representation of our data. This decision was based on (1) modest to no reductions in fit indices with the addition of class 5 and (2) nonsignificant LMR-LRT with the addition of class 5. A limitation of this model was the relatively low entropy (0.65), suggesting that classification accuracy is questionable. Hence, we included covariates in the model to potentially increase entropy and to avoid biased estimates of covariates related to class membership (resulting in an entropy of 0.76 for the final model).

**Figure 1.**
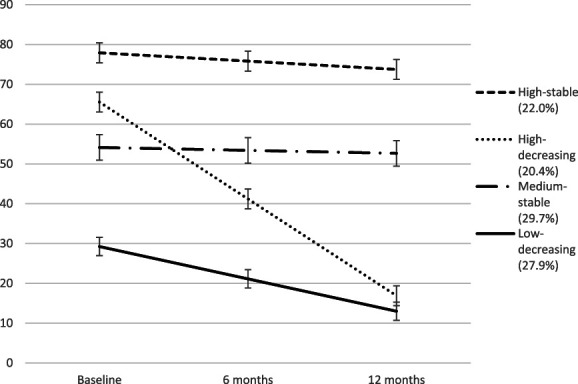
Final model including covariates.

The final four-class model with covariates included can be seen in Figure 1. The final conditional model consisted of a trajectory of initial low functional disability that decreased to an even lower level over time *low-decreasing* (27.9%; intercept [i] = 29.58, slope [s] = −8.24, *P* < 0.001), a trajectory with initial medium functional disability level that remained stable over time *medium-stable* (29.7%; i = 54.25, s = −0.55, *P* = 0.712), a trajectory of high initial functional disability that decreased over time *high-decreasing* (20.4%; i = 66.06, s = −24.55, *P* < 0.001), and finally, a trajectory with high initial symptomatology and only a very small decrease over time *high-stable* (22.0%; i = 78.67, s = −2.51, *P* = 0.039). The entropy of the final model was 0.76.

Using the low-decreasing trajectory as the reference class, we found several significant predictors of membership of one or more of the symptomatic trajectories. Female sex was predictive of membership in the medium-stable trajectory, while higher age increased the likelihood of belonging to the medium-stable or the high-stable trajectories. Baseline pain intensity predicted membership of all 3 symptomatic trajectories, and the same was the case for baseline pain-catastrophizing.

Anxiety at baseline was not related to trajectory membership, while higher level of baseline depression symptoms at baseline predicted membership of all 3 symptomatic classes. Finally, baseline kinesiophobia was related to membership of the high-decreasing and the high-stable trajectories. All odds ratios (ORs) and confidence intervals (CIs) can be seen in Table 3.

**Table 3:** Multinomial logistic regression analysis with the low-decreasing class as reference.

	OR (95% CI)		
	Medium-stable	High-decreasing	High-stable
Sex	1.72 (0.97–3.07)	1.28 (0.53–3.09)	2.41 (0.85–6.79)
Age	1.04 (1.01–1.06)	1.04 (1.00–1.08)	1.08 (1.03–1.14)
Pain intensity	1.46 (1.25–1.70)	1.73 (1.35–1.73)	2.15 (1.65–2.79)
Pain-catastrophizing	1.07 (1.02–1.12)	1.09 (1.03–1.16)	1.12 (1.05–1.20)
Anxiety	0.97 (0.85–1.10)	0.91 (0.77–1.07)	0.98 (0.79–1.20)
Depression	1.45 (1.25–1.67)	1.58 (1.30–1.92)	1.96 (1.61–2.38)
Kinesiophobia	1.02 (0.96–1.09)	1.11 (1.02–1.21)	1.20 (1.06–1.35)

CI, confidence interval; OR, odd ratio.

For targeting of treatment, it can be of great value to be able to distinguish those who start out high and remain high (high-stable) from those who start out high followed by decrease (high-decreasing). Hence, as an extra analysis, we repeated the multinomial logistic regression with the high-decreasing trajectory as reference class. Briefly, we found that members of the high-stable trajectory were older (OR = 1.05, CI = 1.01–1.09), more likely to experience baseline pain intensity (OR = 1.25, CI = 1.00–1.56), and experienced more symptoms of baseline depression (OR = 1.23, CI = 1.10–1.39) compared with members of the high-decreasing group.

The unadjusted mean values and SDs for all outcomes at all time points for the 4 trajectory groups can be seen in Table 4.

**Table 4:** Unadjusted mean values and SDs for trajectories at all time points.

Outcomes	Time	High-stable	High-decreasing	Medium-stable	Low-decreasing
Disability	T1	78.2 (13.7)	71.2 (14.0)	53.3 (16.2)	29.6 (14.7)
	T2	71.9 (16.2)	29.9 (21.9)	49.6 (16.7)	15.2 (13.6)
	T3	75.2 (13.3)	17.9 (12.5)	52.2 (13.1)	13.8 (11.6)
Pain intensity	T1	7.1 (1.8)	6.2 (2.1)	5.6 (1.7)	4.2 (1.9)
	T2	6.4 (2.2)	3.3 (2.4)	5.2 (2.1)	2.7 (2.1)
	T3	6.7 (1.9)	2.7 (2.1)	5.6 (1.9)	2.7 (1.9)
Catastrophizing	T1	30.3 (8.5)	22.8 (8.8)	18.5 (8.0)	12.8 (7.3)
	T2	26.5 (8.9)	12.8 (9.0)	16.0 (8.2)	8.6 (6.9)
	T3	26.0 (9.0)	9.8 (7.2)	15.9 (7.1)	7.6 (6.4)
Anxiety	T1	11.5 (3.6)	8.5 (3.7)	7.3 (3.5)	5.2 (3.1)
	T2	9.8 (4.1)	5.0 (3.6)	5.9 (3.4)	3.4 (2.8)
	T3	10.3 (3.8)	4.2 (3.1)	6.2 (3.4)	3.3 (2.7)
Depression	T1	9.9 (3.5)	6.1 (3.4)	5.0 (2.9)	2.8 (2.1)
	T2	8.9 (3.8)	3.1 (2.9)	4.3 (3.1)	1.7 (2.2)
	T3	9.2 (3.7)	2.2 (2.4)	4.5 (3.1)	1.5 (1.7)
Kinesiophobia	T1	46.0 (5.2)	41.3 (5.5)	37.2 (5.5)	35.6 (5.2)
	T2	43.3 (6.6)	36.5 (6.8)	36.0 (6.1)	31.3 (5.8)
	T3	43.0 (6.4)	34.8 (6.9)	35.4 (5.7)	30.8 (6.7)

## 4. Discussion

Four distinct trajectories of disability were identified: high-stable, high-decreasing, medium-stable, and low-decreasing. As hypothesized, using the low-decreasing trajectory as the reference class, female sex was predictive of membership in the medium-stable trajectory and higher age increased the likelihood of belonging to the medium-stable and the high-stable trajectories. Baseline levels of pain intensity, depressive symptoms, and pain-catastrophizing predicted membership of all 3 symptomatic trajectories. Kinesiophobia was related to both the high-decreasing and the high-stable trajectories. Finally, using the high-decreasing trajectory as reference class, age, baseline pain intensity, and depression were predictors of the high-stable trajectory.

The 4 trajectories identified in the present study are similar to those found by Deyo et al.,^9^ with the exception of the low-moderate trajectory. Interestingly, 2 distinct trajectories were identified, both starting at high levels of disability; however, one trajectory being stable at a high level of disability (high-stable) and the other improving substantially by 75% (high-decreasing). While Deyo et al.^9^ found that only 6.1% recovered by 80%, the present high-decreasing trajectory comprised as many as 20.4% of the total sample. The smaller number belonging to the recovering trajectory in the study by Deyo et al.^9^ may be explained by the patients being on average almost 20 years older than the patients in the present study.

Although multidisciplinary pain rehabilitation is often recommended for chronic LBP, the effect of such interventions has been questioned when taking both effectiveness and costs into consideration.^37^ Hence, multidisciplinary interventions may not suit all, and some subgroups may improve more than others. Indeed, trajectory studies prior to ours have identified distinct subgroups that follow very different trajectories of recovery from both pain intensity^20^ and disability.^9^ On average, improvement in disability scores in relation to LBP is less than the recommended minimal clinical important difference of 30% on the RMDQ.^29^ However, the present study indicates that levels of baseline pain intensity, pain-catastrophizing, and depressive symptoms were associated with lack of recovery. In particular, when compared with the high-decreasing trajectory, baseline pain intensity and comorbid high levels of baseline depressive symptoms were important predictors of the high-stable trajectory. While psychologically augmented physiotherapy has proven to be effective for patients with high levels of pain-catastrophizing and fear-avoidance behaviors,^17^ the finding that depression was associated with lack of recovery indicates that interventions targeting comorbid depression may be needed. Also, if functional disability is more associated with depressive symptoms than pain, treatment of pain symptoms may not be sufficient to restore functioning. This finding is in accordance with the results of a recent systematic review finding depressive symptoms to be an important predictor of lack of recovery from acute and subacute LBP.^32^ Also, of note, the high-stable group newer declined below clinical levels of pain-catastrophizing, anxiety, depression, or kinesiophobia at any time point. For this reason, patients representing the high-stable trajectory may need a more specific psychotherapeutic intervention tailored for comorbid depression and psychological distress. Evidence supports the positive effects of cognitive behavioral interventions for LBP,^33^ and the National Institute for Health and Care Excellence recommends to consider psychological therapies with a cognitive behavioral approach as part of the treatment package.^27^ In addition, early targeting of psychological risk factors is recommended instead of waiting until usual care fails.^30^

Based on the present findings, it is suggested that patients with persistent high levels of pain intensity and disability in combination with high levels of depressive symptoms are further assessed for clinical depression and offered an evidence-based intervention for depression as part of their treatment package if needed.

### 4.1. Limitations

Although similar trajectories of disability have been identified, due to different statistical approaches, our results cannot be directly compared with these. However, the trajectories are in accordance with the previous trajectory studies mentioned. Also, we had only 3 assessments of our cohort; therefore, we could only estimate linear growth, while in reality, the addition of a quadratic could very well have resulted in a better fitting model.

Although only patients with no indication for acute spinal surgical interventions were included in the study, a potential limitation is the lack of information on interventions received during the follow-up period.

## 5. Conclusion

Distinguishing subgroups of patients with different trajectories of recovery underlines the importance of not only classifying patients according to predefined disability categories. In particular, the finding that baseline levels of pain-catastrophizing and depressive symptoms were predictors of the recovery trajectories is promising because they may be targeted by cognitive behavioral therapeutic approaches.

## Disclosures

The authors have no conflict of interest to declare.
